# Molecular features of the ligand-free GLP-1R, GCGR and GIPR in complex with G_s_ proteins

**DOI:** 10.1038/s41421-024-00649-0

**Published:** 2024-02-13

**Authors:** Zhaotong Cong, Fenghui Zhao, Yang Li, Gan Luo, Yiting Mai, Xianyue Chen, Yanyan Chen, Shi Lin, Xiaoqing Cai, Qingtong Zhou, Dehua Yang, Ming-Wei Wang

**Affiliations:** 1 Department of Pharmacology, School of Basic Medical Sciences, Fudan University, Shanghai, China; 2 State Key Laboratory of Chemical Biology, Shanghai Institute of Materia Medica, Chinese Academy of Sciences, Shanghai, China; 3 The National Center for Drug Screening, Shanghai Institute of Materia Medica, Chinese Academy of Sciences, Shanghai, China; 4 Shanghai Institute of Infectious Disease and Biosecurity, Department of Medical Microbiology and Parasitology, School of Basic Medical Sciences, Fudan University, Shanghai, China; 5 Key Laboratory of Medical Molecular Virology (MOE/NHC/CAMS), School of Basic Medical Sciences, Fudan University, Shanghai, China; 6 Research Center for Deepsea Bioresources, Sanya, Hainan China; 7 Department of Chemistry, School of Science, The University of Tokyo, Tokyo, Japan; 8 School of Pharmacy, Hainan Medical University, Haikou, Hainan China

**Keywords:** Electron microscopy, Molecular biology

## Abstract

Class B1 G protein-coupled receptors (GPCRs) are important regulators of many physiological functions such as glucose homeostasis, which is mainly mediated by three peptide hormones, i.e., glucagon-like peptide-1 (GLP-1), glucagon (GCG), and glucose-dependent insulinotropic polypeptide (GIP). They trigger a cascade of signaling events leading to the formation of an active agonist–receptor–G protein complex. However, intracellular signal transducers can also activate the receptor independent of extracellular stimuli, suggesting an intrinsic role of G proteins in this process. Here, we report cryo-electron microscopy structures of the human GLP-1 receptor (GLP-1R), GCG receptor (GCGR), and GIP receptor (GIPR) in complex with G_s_ proteins without the presence of cognate ligands. These ligand-free complexes share a similar intracellular architecture to those bound by endogenous peptides, in which, the G_s_ protein alone directly opens the intracellular binding cavity and rewires the extracellular orthosteric pocket to stabilize the receptor in a state unseen before. While the peptide-binding site is partially occupied by the inward folded transmembrane helix 6 (TM6)–extracellular loop 3 (ECL3) juncture of GIPR or a segment of GCGR ECL2, the extracellular portion of GLP-1R adopts a conformation close to the active state. Our findings offer valuable insights into the distinct activation mechanisms of these three important receptors. It is possible that in the absence of a ligand, the intracellular half of transmembrane domain is mobilized with the help of G_s_ protein, which in turn rearranges the extracellular half to form a transitional conformation, facilitating the entry of the peptide N-terminus.

## Introduction

Class B1 G protein-coupled receptors (GPCRs), consisting of 15 members, have a large N-terminal domain involved in recognition of peptide hormones^1^. Due to their broad physiological functions, class B1 family members are important drug targets for many diseases, including type 2 diabetes, obesity, osteoporosis, migraine, cardiovascular diseases, and short bowel syndrome^2^. As such, understanding the structural underpinnings of receptor activation is of prime interest. The typically assumed GPCR activation mechanism posits that the agonist binding induces conformational changes of the receptor towards active state, resulting in the opening of an intracellular cavity, where G protein binds^3,4^. Structures of all 15 class B1 GPCRs in complex with peptide agonists and G_s_ protein have been determined by cryo-electron microscopy (cryo-EM) in recent years^5^, providing important insights into hormone recognition and receptor activation of this family.

It has been widely accepted that class B1 GPCRs adopt a two-step model for peptide binding and receptor activation^1^. However, several recently determined non-peptidic ligand-bound structures of class B1 GPCRs have uncovered intracellular ligand-binding pockets. A prototypical example is PCO371, which could bind within the highly conserved intracellular pocket, directly and selectively interact with G_s_ protein, activating 7 out of 15 family members^6,7^. In addition, compound 2, an ago-positive allosteric modulator (ago-PAM), has been found to covalently bond to the intracellular side of transmembrane helix 6 (TM6) and allosterically induce insertion of the N-terminus of extracellular domain (ECD) into the orthosteric binding site of glucagon-like peptide-1 receptor (GLP-1R), thus triggering G_s_-biased activation^8^. Meanwhile, GPCRs can also be activated in the absence of a ligand causing basal signaling^9,10^.

GLP-1R, glucagon receptor (GCGR) and glucose-dependent insulinotropic polypeptide receptor (GIPR) are validated therapeutic targets for type 2 diabetes and obesity^11–13^. There exist a number of crystal and/or cryo-EM structures of them in inactive, intermediate and active states revealing key information about their interactions with various ligands^2,8,14,15^, including dual or triple agonist-bound GLP-1R, GCGR or GIPR–G_s_ complexes^16–18^. Although these structures provide molecular insights into diverse activation mechanisms of these three receptors, it remains unclear whether an extracellular agonist is required for G protein coupling and downstream signal transduction.

Here, we report the cryo-EM structures of GLP-1R, GCGR, and GIPR in complex with G_s_ protein in the absence of any ligand. Based on multi-perspective structural analyses, we present a transitional state, in which G_s_ protein directly interacts with the inactive receptor prior to ligand binding and opens the intracellular cavity by breaking the conserved HETY network (H^2.50b^–E^3.50b^–T^6.42b^–Y^7.57b^) and cytoplasmic polar network (R^2.46b^–R^6.37b^–N^7.61b^–E^8.49b^). G_s_ protein thus stabilizes the receptor in a distinct extracellular conformation until agonist binding to the orthosteric site. This process tightens the extracellular pocket and intracellular region allowing G_s_ protein coupling and full activation of the receptor.

## Results

### Structures of ligand-free receptor–G_s_ complexes

**Fig. 1:**
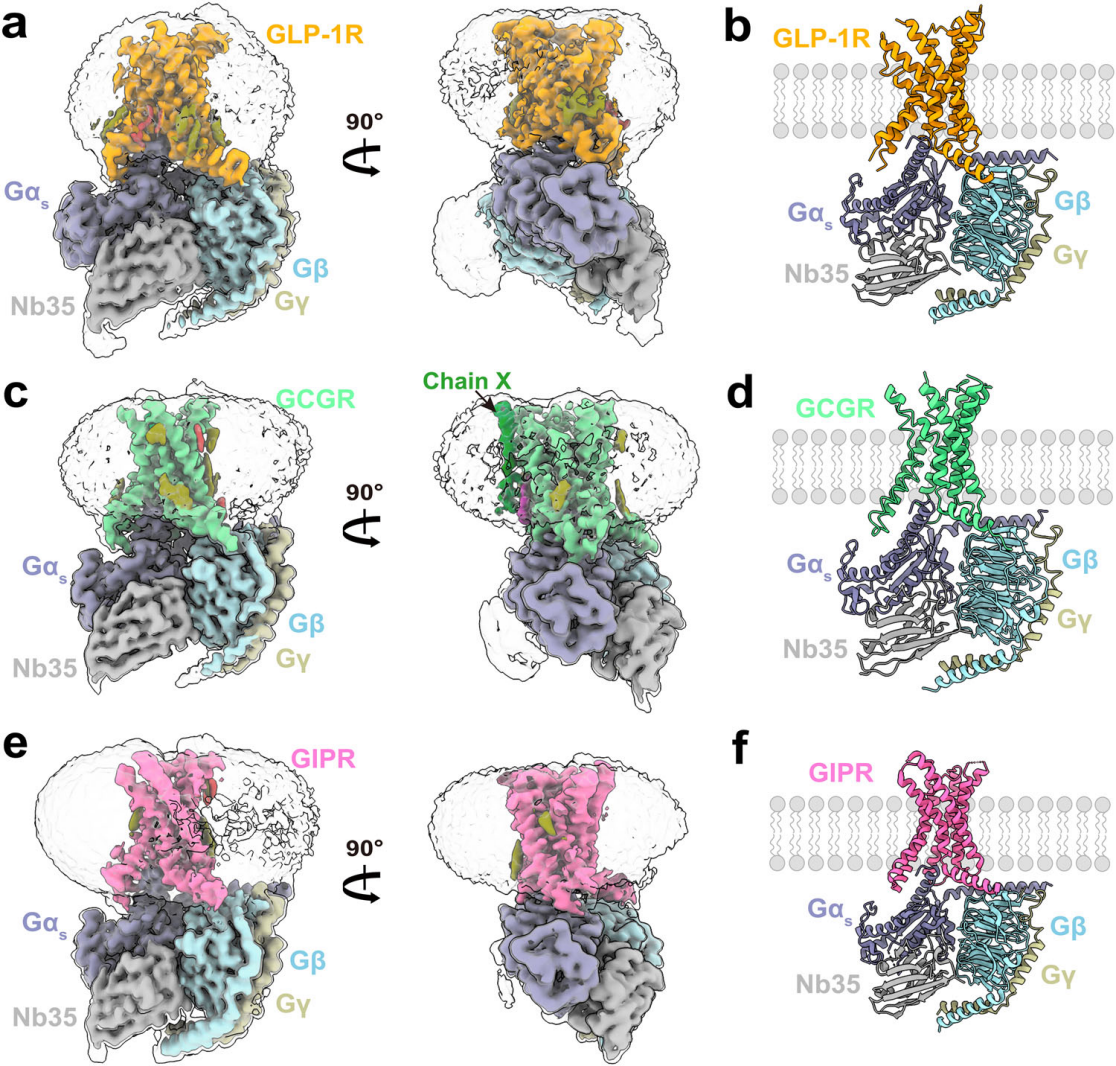
Cryo-EM structures of the ligand-free GLP-1R, GCGR, and GIPR in complex with G_s_ proteins. **a,** **c**, **e** Cryo-EM density maps of the GLP-1R–G_s_ (**a**), GCGR–G_s_ (**c**), and GIPR–G_s_ (**e**) complexes are shown from two viewpoints. GLP-1R, GCGR, GIPR, Gα_s_, Gβ, Gγ, and Nb35 are shown in orange, pale green, hot pink, slate gray, powder blue, khaki and light gray, respectively. The maps reveal several lipid densities around the receptors consistent with the shape of cholesterol (olive), palmitate (brown) or phosphatidylinositol 4,5-bisphosphate (purple). There is a strong unassigned cylindrical density in the shape of helix (green) dropping down along the TM4 and TM5 of GCGR, tentatively named as chain X (**c**). **b**, **d**, **f** Structural models of the GLP-1R–G_s_ (**b**), GCGR–G_s_ (**d**) and GIPR–G_s_ (**f**) complexes are constructed from the respective cryo-EM maps and shown in ribbon.

The cryo-EM structure of ligand-free GLP-1R–G_s_ complex (Supplementary Fig. S1a) was determined at an overall resolution of 2.54 Å (Fig. 1a; Supplementary Figs. S2a and S3a). Structural superimposition shows that this structure is almost identical to that bound by the ago-PAM compound 2 with a Cα root mean square deviation (RMSD) of 0.53 Å (Supplementary Fig. S4), indicating a newly discovered conformational state beyond those of antagonist-bound, apo without G protein, agonist-bound and agonist-bound plus G protein. Following a similar protocol (Supplementary Fig. S1b, c), three-dimensional (3D) structures of the ligand-free GCGR–G_s_ and GIPR–G_s_ complexes were obtained at global resolutions of 2.70 Å and 2.86 Å, respectively (Fig. 1c, e; Supplementary Fig. S2b, c). They display canonical structural features of the GPCR–G protein complex and no extra density was observed in the transmembrane domain (TMD) binding pocket, confirming the absence of any ligand (Fig. 1). The density maps clearly exhibit the backbones and most side chains of TMs, extracellular loops (ECLs), intracellular loops (ICLs), the amphipathic helix 8 (H8), and three G_s_ subunits (Fig. 1; Supplementary Fig. S3). Moreover, the interface residues between the receptor and α5 helix of G_s_ (GαH5) were solved (Supplementary Figs. S3 and S5) and no ECD density was seen in the three maps (Fig. 1), suggesting that the receptors may not require a specific ECD conformation to engage G_s_. Interestingly, an electron density of cylindrical helix shape was found in the GCGR membrane-facing surfaces of TM4 and TM5 that were omitted from modeling due to sequence uncertainty (Fig. 1c).

### G_s_ protein coupling by ligand-free receptors

**Fig. 2:**
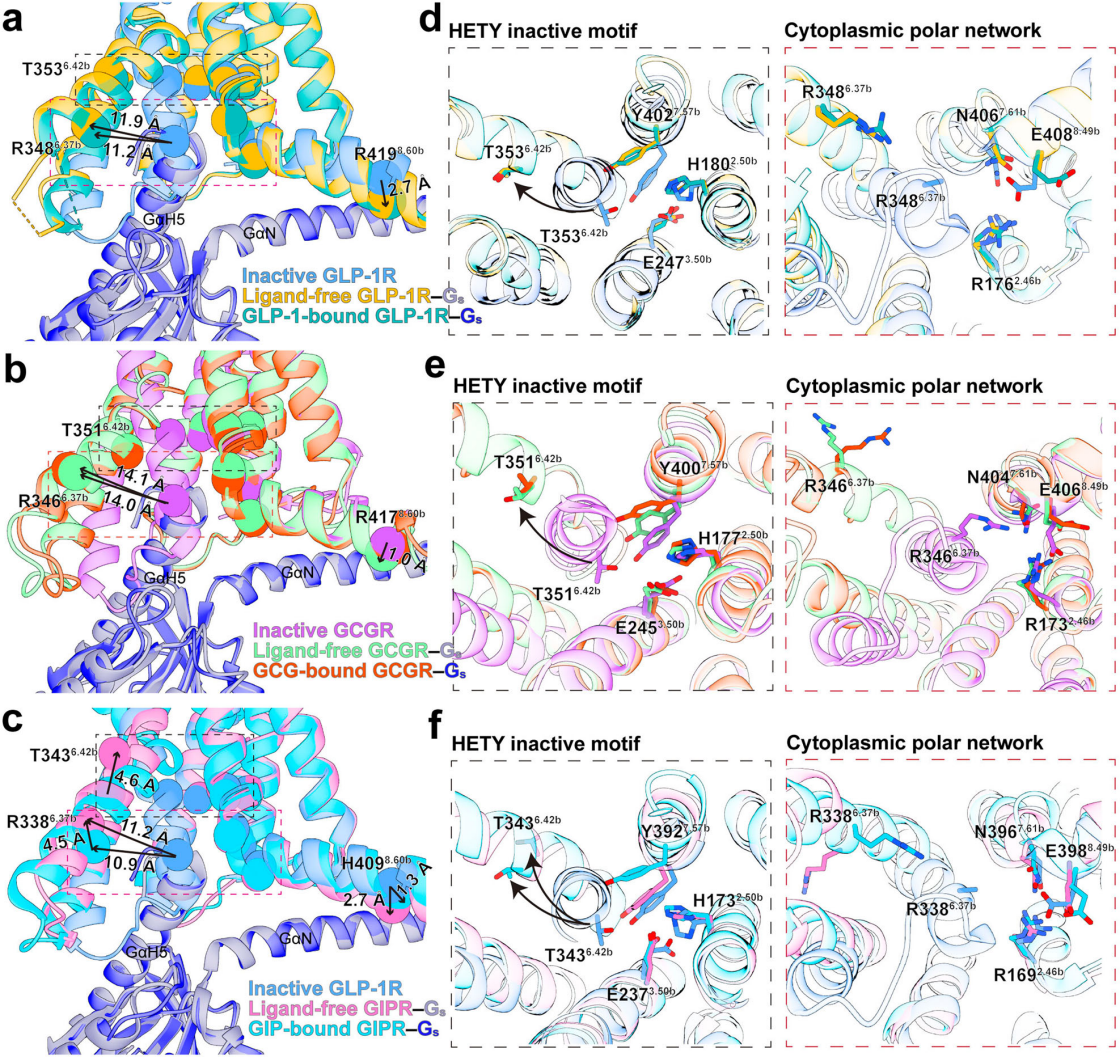
General features of the ligand-free receptors by G_s_ coupling. **a**–**c** Comparison of ligand-free and peptide-bound GLP-1R (**a**), GCGR (**b**) and GIPR (**c**) with inactive receptor structures reveals a similar G_s_ coupling interface in the absence or presence of an agonist. Black arrows show the movements of TM6 and H8 by indicating the distances of Cα atoms of T^6.42b^, R^6.37b^, and R/H^8.60b^ residues. **d**–**f** Structural comparison of the TMD bundles of GLP-1R (**d**), GCGR (**e**) and GIPR (**f**) indicates the requirement of G_s_ protein for receptor activation. Conformational changes are shown for the conserved HETY inactive motif (H^2.50b^–E^3.50b^–T^6.42b^–Y^7.57b^) and cytoplasmic polar network (R^2.46b^–R^6.37b^–N^7.61b^–E^8.49b^). Black arrows indicate the hallmark conformational changes of TM6. The Gβ and Gγ subunits are omitted for clarity. PDB IDs: 6LN2 (inactive GLP-1R), 5XEZ (inactive GCGR), 6X18 (GLP-1-bound GLP-1R), 6LMK (GCG-bound GCGR), and 7DTY (GIP-bound GIPR). The position of N^7.61b^ in **d**, **e** and **f** refers to N^8.47b^ in GPCRdb numbering.

The three ligand-free receptors interact with G_s_ proteins similarly to those bound by endogenous agonists, suggesting a common mechanism for G protein coupling (Fig. 2). It appears that G_s_ is able to induce a consensus kink at the middle of TM6 without an agonist. Subsequently, an outward shift of the cytoplasmic end of TM6 (~11.9 Å in GLP-1R (measured at the Cα of R348^6.37b^), 14.1 Å in GCGR (measured at the Cα of R346^6.37b^) and 10.9 Å in GIPR (measured at the Cα of R338^6.37b^)) renders the receptor to form a cavity for G_s_ coupling (Fig. 2). However, the position of the cytoplasmic end of TM6 in the inactive state (either antagonist-bound or apo without G protein) structures is incompatible with the insertion of the C-terminus of GαH5 (Fig. 2a–c), indicating that G_s_ coupling is necessary for the kink of TM6. While TM6 conformations are identical among ligand-free and agonist-bound GLP-1R and GCGR structures, the movement of TM6 at intracellular region of GIPR is significantly different (4.5 Å measured at the Cα of R338^6.37b^ and 4.6 Å measured at the Cα of T343^6.42b^) (Fig. 2c), pointing to a receptor-dependent structural feature.

It is known that two highly conserved polar networks at the cytoplasmic face of class B1 GPCRs, the HETY network (H^2.50b^–E^3.50b^–T^6.42b^–Y^7.57b^) and the cytoplasmic polar network (R^2.46b^–R^6.37b^–N^7.61b^–E^8.49b^), lock the base of the receptor in a closed conformation^19^. G protein binding eliminates the interactions between T^6.42b^ or R^6.37b^ and other three residues within the respective motif, releasing them from tightly packing constraints to participate in G_s_ coupling (Fig. 2). With respect to ligand-free GLP-1R, the conformational transition induced by G_s_ coupling resulted in breakup of these two networks to release R176^2.46b^ in the TM2–ICL1 juncture, N406^7.61b^ and E408^8.49b^ in the TM7–H8 juncture as well as Y402^7.57b^ and H180^2.50b^ in the TMD core, to facilitate G_s_ coupling (Fig. 2d). Superimposition of receptor–G_s_ complex structures with or without agonist reveals that, although there are limited differences in the overall G_s_ conformation, the ligand-free receptors make less interactions with Gα_s_ Ras and Gβγ domains (Supplementary Figs. S5–S7). For GLP-1R and GCGR, the key Y391^GαH5^ side chain is hosted in a pocket defined by R^2.46b^, Y^3.53b^ and L^3.54b^, while L393^GαH5^ and L394^GαH5^ interact with a set of residues located in proximity to the polar networks, such as L^3.54b^, I^5.57b^, and L^6.43b^, a feature shared by ligand-bound and ligand-free structures (Supplementary Fig. S5a, b). For GIP-bound GIPR, the C-terminal residues of GαH5 (L388, Y391, L393, and L394) insert into a hydrophobic pocket (formed by L241^3.54b^, L244^3.57b^, L321^5.61b^, and L325^5.65b^), and L394^GαH5^ makes one hydrogen bond with the side chain of R338^6.37b^. This phenomenon was not observed in the ligand-free GIPR (Supplementary Fig. S5c), indicating that a conformational rearrangement occurs in the intracellular side upon GIP binding. Of note, E392^GαH5^ switches toward the TMD core to form multiple hydrogen bonds with L401^7.56b^, N406^7.61b^, and N407^8.48b^ in the GLP-1-bound GLP-1R (Supplementary Fig. S5a) and flips downward to make polar interactions with positively charged residues such as K^8.48b^ and N^7.61b^ in the peptide-bound GCGR and GIPR (Supplementary Fig. S5b, c). These polar contacts are significantly reduced in the ligand-free receptor–G_s_ complexes, likely due to the rotation of the side chain of Y^7.57b^. Following peptide binding, Y^7.57b^ shifts toward G_s_ protein-binding face in GCGR and GIPR, while stands still in the case of GLP-1R (Fig. 2d–f). Previous study demonstrated that removal of the hydroxyl group of Y400^7.57b^ in GCGR led to a 40% decrease in maximal cAMP signaling elicited by glucagon^20^, suggesting a critical role of this conformational change.

Besides TMs, ICL2 and ICL3 of the ligand-free receptors contribute weaker contacts to maintain the receptor–G_s_ interface compared to those bound by agonist (Supplementary Figs. S6, S7). Unlike the upward conformation in the ligand-free structures, F257^ICL2^ of GLP-1-bound GLP-1R switches downward and forms intensive stacking interactions with R380^GαH5^, F376^GαH5^, and H41^GαN^. Meanwhile, H8 moves closer to Gβ and makes intensive hydrophobic interactions with V307, A309, and G310 in Gβ subunit (Supplementary Fig. S6). Similar reduced contacts are observed at the ICL2 of GCGR and GIPR, where E260^ICL2^ of GCGR as well as S251^ICL2^ and E253^ICL2^ in GIPR make negligible contacts with G protein in the ligand-free structures. Furthermore, the hydrogen bonds between R380^GαH5^ and ICL2 with the backbone atoms of L245^3.58b^, V246^3.59b^, L247^3.60b^, and V248^ICL2^ are also reduced in the ligand-free GIPR structure (Supplementary Fig. S6). The polar residues S251 and E253 in ICL2 form two hydrogen bonds with K34^GαN^ and Q35^GαN^, and H8 forms hydrogen bonds with Gβ (E402^8.53b^–R164^ICL1^–D312^Gβ^), but these interactions are not observed in the ligand-free GIPR structure (Supplementary Fig. S6). For ICL3, the TM5–ICL3 region only has a limited density in both GLP-1-bound and ligand-free GLP-1R structures, but is clearly visible for those of GCGR and GIPR (Supplementary Fig. S7). In the agonist-bound structures, GαN helices interact with ICL3 through Y385^GαH5^, but this interaction is not found in the ligand-free GLP-1R and GCGR structures and greatly reduced in that of GIPR (Supplementary Fig. S7).

### TM packing induced by G_s_ coupling

**Fig. 3:**
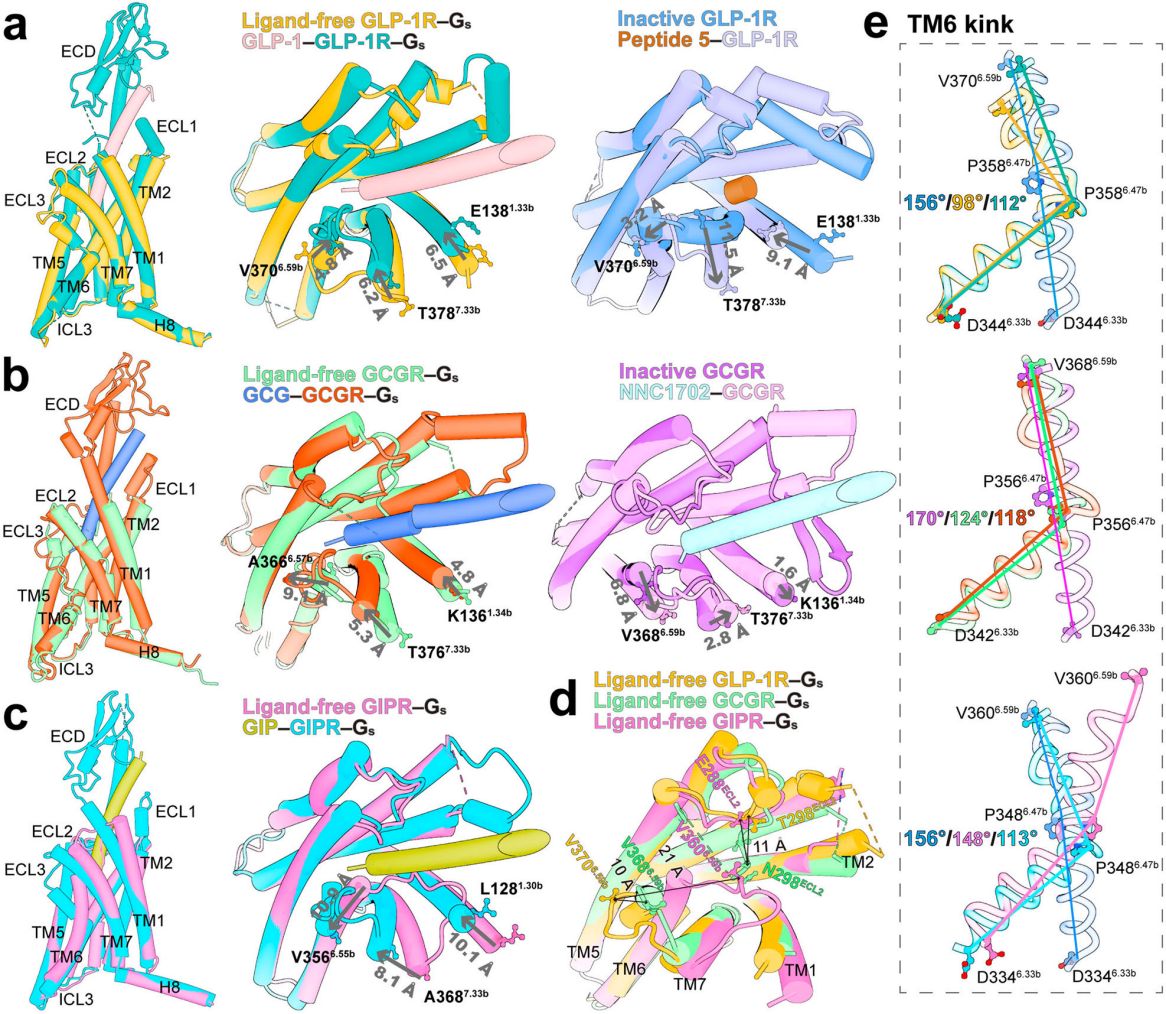
G_s_ coupling induced rearrangements in the extracellular portion of TMD. **a** Structural superimposition of ligand-free and GLP-1-bound GLP-1R–G_s_ (PDB: 6X18) complexes from side and top views, as well as inactive GLP-1R (PDB: 6LN2) and peptide 5-bound GLP-1R (PDB: 5NX2) from top view, shows different conformational changes induced by agonist binding with or without G_s_ protein coupling. Gray arrows indicate the movements of TM1, TM6 and TM7 measured by the Cα atoms of E138^1.33b^, V370^6.59b^ and T378^7.33b^, respectively. **b** Structural superimposition of ligand-free and GCG-bound GCGR–G_s_ (PDB: 6LMK) complexes from side and top views, as well as inactive GCGR (PDB: 5XEZ) and NNC1702-bound GCGR (PDB: 5YQZ) from top view, shows different conformational changes induced by agonist binding with or without G_s_ protein coupling. Gray arrows indicate the movements of TM1, TM6, and TM7 measured by the Cα atoms of K136^1.34b^, V368^6.59b^, and T376^7.33b^, respectively. **c** Structural superimposition of ligand-free and GIP-bound GIPR–G_s_ (PDB: 7DTY) complexes from side and top views shows a unique conformational change in the extracellular region induced by GIP binding. Gray arrows indicate the movements of TM1, TM6 and TM7 measured by the Cα atoms of L128^1.30b^, V356^6.55b^, and A368^7.33b^, respectively. **d** Superimposition of ligand-free GLP-1R, GCGR and GIPR structures reveals dynamics of the ECLs. The conformational differences are indicated by two-way arrows. **e** Structures of ligand-free and endogenous ligand-bound receptors are superimposed onto the inactive GLP-1R or GCGR structure, showing different TM6 kink conformations. The kink angle is shown among the Cα atoms of V^6.59b^–P^6.47b^–D^6.33b^ in TM6. G_s_ proteins are omitted for clarity.

Superimposition of the ligand-free and peptide-bound structures shows that the intracellular half of TMD aligns well, while the extracellular half is structurally divergent and exhibits a receptor-specific conformation in the absence of ligand (Fig. 3a–c). In addition, TM6 kink is the most significant structural feature of ligand-free G_s_-coupled receptors distinguished from that of inactive or intermediate states (Supplementary Fig. S8a, b). For GLP-1R, G_s_ coupling induces a significant outward movement of the extracellular portion of TM bundles (Supplementary Fig. S8a). Upon GLP-1 binding, 6.5 Å, 4.8 Å, and 6.2 Å inward movements of the extracellular portion of TM1, TM6 and TM7 (measured at the Cα atoms of E138^1.33b^, V370^6.59b^, and T378^7.33b^) were seen, respectively. However, in the absence of G_s_, the TM1 movement from inactive state to agonist peptide 5-bound state is larger (9.1 Å), while TM6 and TM7 shift outward by 3.2 Å and 11.5 Å, respectively (Fig. 3a). These conformational rearrangements of the ligand-free GLP-1R are in general consistent with those observed in the compound 2-bound GLP-1R–G_s_ structure^8^ and the apo-state GLP-1R obtained from structure determination of tirzepatide-bound GLP-1R–G_s_ complex^21^ (Supplementary Fig. S8c), except that in our new structure, ECL3 splays out and causes a sharper TM6 kink distinct from the GLP-1-bound active structure as seen from the kink angle (98° and 112° for the ligand-free and GLP-1-bound GLP-1R–G_s_ structures, respectively, measured at the Cα atoms of D344^6.33b^–P358^6.47b^–V370^6.59b^) (Fig. 3e).

Unlike GLP-1R, GCGR in complex with G_s_ has an almost identical kink angle of TM6 (~120° measured at the Cα of D342^6.33b^–P353^6.47b^–V358^6.59b^) regardless of peptide binding or not (Fig. 3e), indicative of a relatively stable TM6–ECL3–TM7 conformation. In terms of the extracellular portion, TM1 and TM7 in the NNC1702-bound GCGR slightly move outward relative to the inactive-state structure. However, in the presence of G_s_, GCG binding causes the extracellular portions of TM1 and TM7 to move inward by 4.8 Å (measured at the Cα of K136^1.34b^) and 5.3 Å (measured at the Cα of T376^7.33b^), respectively, accompanied by a 9.1 Å (measured at the Cα of A366^6.57b^) outward movement of TM6 (Fig. 3b). The ligand-free GIPR displays the most profound structural feature of TM packing, in which the extracellular portion of TM6 and ECL3 fold inward to occupy the TMD pocket. Consequently, the kink in TM6 adopts a unique angle (148° measured at the Cα of D334^6.33b^–P348^6.47b^–V360^6.59b^) different from those of GIP-bound GIPR (113°) and peptide-free GLP-1R and GCGR (Fig. 3e). Upon GIP binding, the extracellular tip of TM6 moves outward by 10.9 Å (measured by the Cα distance of V356^6.55b^) to facilitate the insertion of GIP. Meanwhile, the extracellular halves of TM1 and TM7 move toward the orthosteric pocket by 10.1 Å (measured at the Cα of L128^1.30b^) and 8.1 Å (measured at the Cα of A368^7.33b^), respectively (Fig. 3c). These data imply that G_s_ coupling rewires the extracellular halves of TMs in a receptor-dependent manner.

At the residue level, the central polar network (K/R^2.60b^–N^3.43b^–H^6.52b^–Q^7.49b^) located below the peptide-binding pocket and PxxG (P^6.47b^–L^6.48b^–L^6.49b^–G^6.50b^) motif where the TM6 sharp kink occurs directly or indirectly participates in peptide recognition by switching from loose (ligand-free structure) to compact packing (peptide-bound structure) to tighten the TMD orthosteric pocket (Supplementary Fig. S9). Upon peptide binding, the central polar network is not reconstructed in GLP-1R, but most residues change their conformations and partially pull out of the orthosteric pocket in the case of GCGR. Conformational divergence around the peptide-binding pocket is more evident in GIPR, due to the steric clashes of the bent TM6–ECL3 (Supplementary Fig. S9b). The ligand-free GLP-1R and GCGR deform the PxxG motif in a manner similar to the agonist-bound structures, while the ligand-free GIPR prefers a moderately kinked TM6 that is significantly different from the fully active structures. The inward rotation of the PxxG motif in GLP-1R and GCGR is further stabilized by hydrogen bonds with TM5 (G^6.50b^–N^5.50b^) and TM7 (P^6.47b^–Q^7.49b^), as well as hydrophobic contacts with F^5.54b^ and V^7.53b^, a phenomenon not observed in the ligand-free GIPR structure (Supplementary Fig. S9c).

### Conformational flexibility of the ECLs

Structural superimposition of the ligand-free G_s_-coupled GLP-1R, GCGR and GIPR with those bound by endogenous peptides reveals that the ligand-binding pocket of ligand-free receptor is partially occupied by GCGR ECL2 or GIPR ECL3 but not the ECLs of GLP-1R. In all the three structures, the densities of most ECL1 residues are invisible, whereas ECL2 and ECL3 are well-defined in the EM maps of GLP-1R and GIPR, and largely solved in GCGR except one short segment (D299–G302) (Supplementary Fig. S3). The peptide-binding pocket in the ligand-free GLP-1R exhibits a more opened conformation than those of the other two receptors. This is because of a large outward movement of GLP-1R ECL3 (~10 Å measured at the Cα of V370^GLP-1R^–V368^GCGR^, and 21 Å measured at the Cα of V370^GLP-1R^–V360^GIPR^), despite that the ECL2 conformation is well maintained (Fig. 3d).

**Fig. 4:**
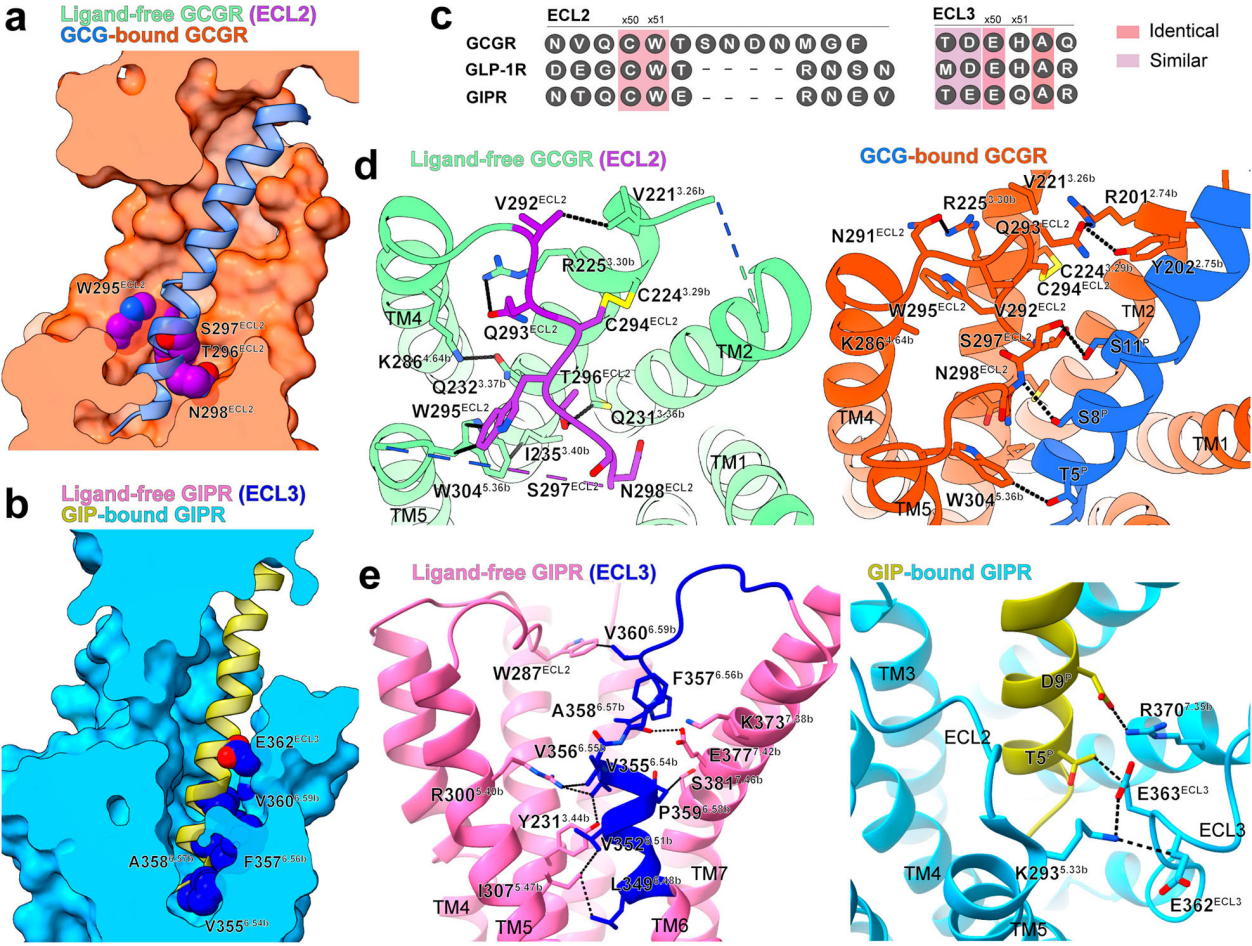
ECL2 of GCGR and ECL3 of GIPR occupy the classic orthosteric binding site for peptide. **a** Superimposition of ligand-free and GCG-bound GCGR structures reveals that the GCG-binding pocket is partially occupied by inward moved ECL2 upon G protein coupling. GCG-bound GCGR (PDB: 6LMK) is shown in surface representation. ECL2 residues of ligand-free GCGR are shown in sphere. **b** Superimposition of ligand-free and GIP-bound GIPR structures reveals that the GIP-binding pocket is partially occupied by inward moved TM6–ECL3 juncture upon G protein coupling. GIP-bound GIPR (PDB: 7DTY) is shown in surface representation. Residues in the TM6–ECL3 juncture of ligand-free GIPR are shown in sphere. **c** Sequence alignment of ECL2 and ECL3 of GCGR, GLP-1R and GIPR. **d** Magnified view of the ECL2 within the orthosteric binding pocket (left panel) and its interaction with GCG (right panel). **e** Magnified view of the TM6–ECL3 juncture in the orthosteric binding pocket (left panel) and its interaction with GIP (right panel). Key interacting residues and their contacts are shown as sticks and dashed lines, respectively.

Unlike GLP-1R, N291–N298 segment of ECL2 in the ligand-free GCGR stretches toward the TMD core to partially occupy the orthosteric pocket, which overlaps with the N-terminal segment (T5^P^–S8^P^) of GCG (Fig. 4a). This unique ECL2 conformation may result from interactions with chain X (Supplementary Fig. S10a). Superimposition of the ligand-free and GCG-bound GCGR structures reveals that five residues in the TM4, TM5 and ECL2 spatially clash with the cryo-EM density of chain X, including four phenylalanine (F278^4.56b^, F289^4.67b^, F303^ECL2^, and F309^5.41b^) and one isoleucine I306^5.38b^ (Supplementary Fig. S10a). To avoid this steric hindrance, several aromatic amino acids shift inwards, pushing ECL2 to occupy the peptide-binding pocket (Supplementary Fig. S10b). Specifically, residues F289^4.67b^, F303^ECL2^, and F309^5.41b^ flip away from chain X–GCGR interface to create room for chain X binding. This conformational change is propagated to W295^ECL2^, W304^5.36b^, and W305^5.37b^, pushing them to rotate toward the central pore and inducing W282^4.60b^ to shift outward (Supplementary Fig. S10c). Consequently, this “F–W switch” in the vicinity of chain X creates steric clashes with ECL2 and facilitates its stretched conformation. In the ligand-free GCGR, N291–N298 segment of ECL2 points to the TMD core like a “latch”, and is stabilized by hydrogen bonds (N291^ECL2^–R225^3.30b^–Q293^ECL2^), hydrophobic contacts via V221^3.26b^, M231^3.36b^ and Q232^3.37b^ and a conserved disulfide bond (C294^ECL2^–C224^3.29b^) (Fig. 4d). In addition, the movements of TM4 and TM5 causes the formation of two additional residue pairs under the ECL2, one hydrogen bond (Q232^3.37b^–K286^4.64b^) and several hydrophobic contacts (I235^3.40b^–W304^5.36b^), to lock ECL2 in the orthosteric pocket. However, in the GCG-bound structure, ECL2 mainly interacts with the peptide N-terminus through D299–G302 segment (Fig. 4d). Recent studies have shown that several class A orphan GPCRs are self-activated by ECL2 that directly penetrates to the orthosteric binding pocket, such as GPR21^22^, GPR52^23^, GPR17^24^, GPR161 and GPR61^25^. Although the ECL2 of GCGR partially overlaps with the peptide-binding site, the majority of which is likely disordered owing to the missing cryo-EM density, suggesting its conformational flexibility and non-built-in agonist nature.

Instead of ECL2, TM6–ECL3 of ligand-free GIPR folds toward the TMD core and occupies the orthosteric pocket (Fig. 4e; Supplementary Fig. S11). Specifically, TM6–ECL3 is clasped by TM5 and TM7 and stabilized by hydrophobic interactions with TM3, TM5 and ECL2 via L349^6.48b^, V352^6.51b^, V355^6.54b^, P359^6.58b^ and V360^6.59b^, and by polar contacts with TM1, TM3 and TM7 via E354^6.53b^, F357^6.56b^, E363^ECL3^, Q364^ECL3^ and A365^ECL3^. In the GIP-bound GIPR structure, the ECL3 is stabilized by two polar contacts (T5^P^–E363^ECL3^ and D9^P^–R370^7.35b^) (Fig. 4e). W287^ECL2^ orientates its side chain from the TM3–TM4 crevice in the GIP–GIPR–G_s_ structure to occupy the orthosteric site in the ligand-free structure, reflecting the engagement of GIPR ECL2 in the closure of TMD ligand-binding pocket.

### Activation-related conformational changes

**Fig. 5:**
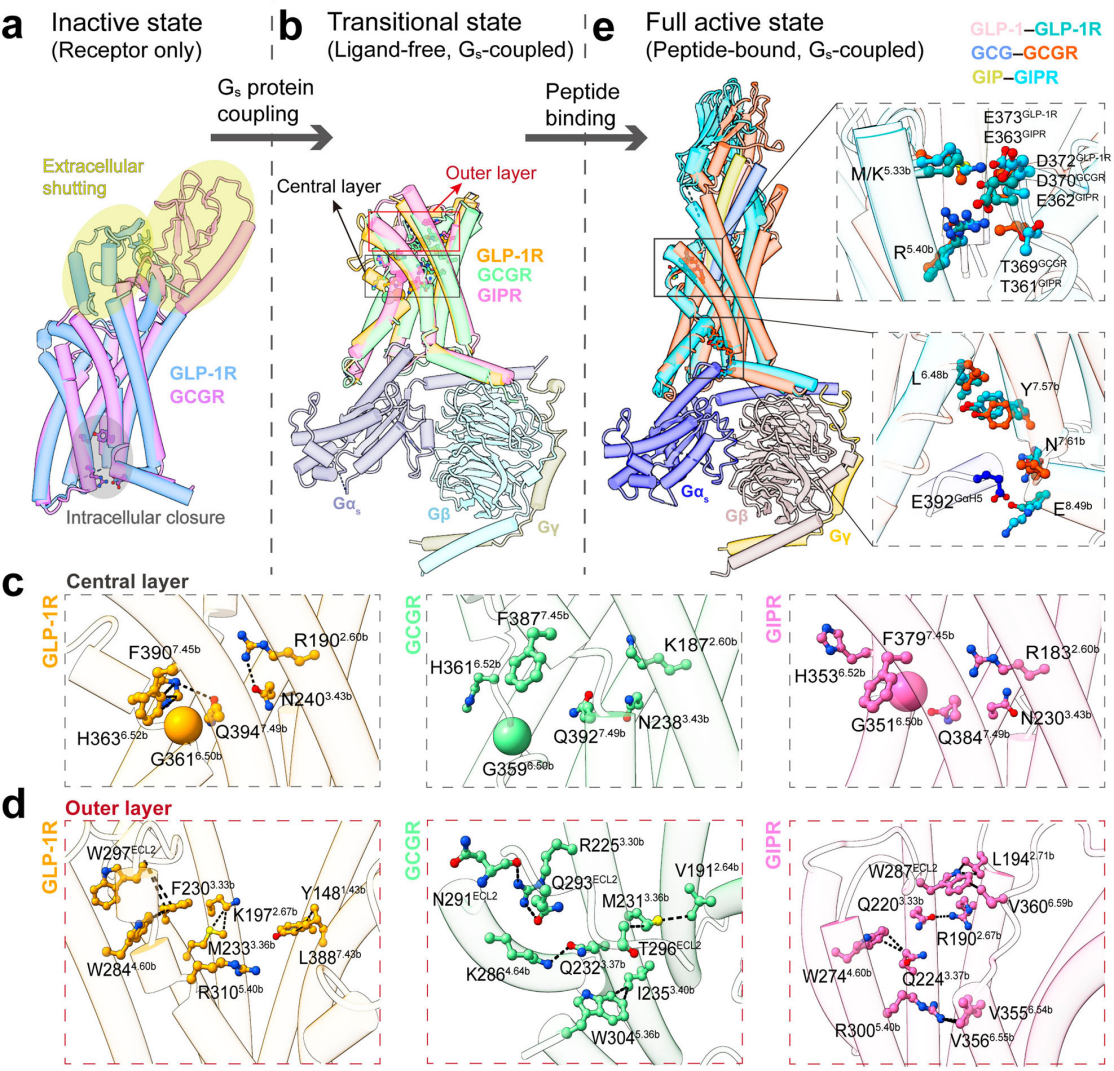
Mechanistic implication of ligand-free and G_s_-coupled GLP-1R, GCGR and GIPR complex structures. **a** Superimposition of inactive GLP-1R and GCGR structures shows an extracellular shutting conformation (yellow shadow) and intracellular closed conformation (gray shadow), thus intercepting peptide binding and G_s_ coupling. **b** Superimposition of ligand-free GLP-1R, GCGR and GIPR structures shows that G_s_ coupling directly causes the formation of an intracellular cavity and stabilizes the intracellular half of TMD, whereas the extracellular portion of the receptor is stabilized in the central and outer layers. **c**, **d** Magnified views of different contacting modes in the central (**c**) and outer (**d**) layers of GLP-1R, GCGR and GIPR. Key interacting residues are shown as ball-and-sticks, and the interactions are shown as dashed lines. **e** Superimposition of endogenous ligand-bound GLP-1R, GCGR and GIPR structures shows that peptide binding stabilizes the TM6–ECL3–TM7 conformation and rewires G_s_ protein. The right panel shows magnified views of the M/K^5.33b^–R^5.40b^–T/D/E^ECL3^ and L^6.48b^–Y^7.57b^–N^7.61b^–E^8.49b^–E392^GαH5^ interfaces. Key interacting residues are shown as ball-and-sticks. PDB IDs: 6LN2 (inactive GLP-1R), 5XEZ (inactive GCGR), 6X18 (GLP-1-bound GLP-1R), 6LMK (GCG-bound GCGR) and 7DTY (GIP-bound GIPR). The position of N^7.61b^ in **e** refers to N^8.47b^ in GPCRdb numbering.

Inspecting conformational changes of highly conserved residues reveals: (i) several distinct fastening pieces within the central and outer layers that stabilize the receptor in a transitional state; and (ii) similar contacts within the ECL3–TM5 interface and intracellular cavity that respond to peptide binding and may transmit extracellular stimuli to G_s_ protein (Fig. 5). In the absence of agonist and G_s_ protein, the receptors are locked in an inactive state, posing an extracellular shutting conformation through interactions between ECD and ECLs and an intracellularly closed conformation through interactions within conserved HETY and cytoplasmic polar networks, thereby preventing peptide binding and G_s_ coupling (Fig. 5a). G_s_ coupling will break the bottom polar networks, interfere with movement of the extracellular portions and render the receptor to a transitional state (Fig. 5b). Overall, the transitional conformation is close to the fully active ensemble in the intracellular half, but the extracellular half, especially the peptide-binding site, is stabilized differently. In the ligand-free GLP-1R, G_s_ coupling rearranges several residues located above the PxxG motif, which form hydrogen bonds (Q^7.49b^–G^6.50b^ and N^3.43b^–R^2.60b^) and π stacking (H^6.52b^–F^7.45b^) at the central layer (Fig. 5c), thus maintaining the TMD and ECLs in a conformation close to the active state. However, these interactions are disrupted by marked shifts of TM1, TM6 and TM7 in the ligand-free GCGR and GIPR. Located above the central network is a cluster of hydrophobic packing interactions, stabilizing the orthosteric site in different conformations. For GLP-1R, residues Y148^1.43b^, F230^3.33b^, M233^3.36b^ and R310^5.40b^ partially occupy the orthosteric stie and form hydrophobic interactions with K197^2.67b^, W297^ECL2^ and L388^7.43b^, as well as π stacking with W284^4.60b^. However, the ligand-free GCGR mainly composes the structural feature in the TM4–ECL2–TM5 region, where hydrophobic interactions (W304^5.36b^–I235^3.40b^ and M231^3.36b^–V191^2.64b^) and hydrogen bonds (Q232^3.37b^–K286^4.64b^ and Q293^ECL2^–N291^ECL2^/R225^3.30b^) form. Notably, in the ligand-free GIPR, the TM6–ECL3 juncture is sandwiched by the TM5–ECL2 juncture and the extracellular portion of TM7, weaving an intensive interaction network by a hydrogen bond between Q220^3.33b^ and R190^2.67b^, and several hydrophobic contacts (R300^5.40b^–V355^6.54b^/V356^6.55b^, W274^4.60b^–Q224^3.37b^, and W287^ECL2^–L194^2.71b^/V360^6.59b^) (Fig. 5d).

Upon peptide binding, M/K^5.33b^ and R^5.40^ switch toward ECL3 to tighten the orthosteric pocket by interactions within the TM5–ECL3 interface, which was observed in all three structures (Fig. 5e). The distinct fastening pieces within the central and outer layers of the ligand-free receptors are also reshaped to a similar conformation, stabilizing the active state by interactions with peptide N-terminus as reported previously^15,26,27^. Obviously, agonist binding can enhance G_s_ coupling, as seen from the increased interface area and strengthened interactions (Supplementary Figs. S5–S7), suggesting that peptide binding not only tightens the pocket, but also transmits extracellular signal to G_s_ protein. Structural comparison shows that the conserved residues L^6.48b^, Y^7.57b^, N^7.61b^ and E^8.49b^ relocate E392^GαH5^ to stabilize G_s_ protein coupling (Fig. 5e). Thus, despite the different conformational changes in the orthosteric pocket, activation signal is transduced from the extracellular to intracellular sides through L^6.48b^–Y^7.57b^–N^7.61b^–E^8.49b^–E392^GαH5^ motif, where L^6.48b^ pushes Y^7.57b^ to a similar position, transmitting to N^7.61b^ and E^8.49b^ to respond to G_s_ residue E392 (Fig. 5e), thus fully activating the receptor. Collectively, upon agonist binding, the receptor in a transitional state may rapidly initiate residue rearrangement without the need of large TM movement to activate signal transduction.

## Discussion

GLP-1R, GCGR and GIPR are essential regulators of glucose homeostasis and therapeutic targets for type 2 diabetes and obesity. It has been widely accepted that class B1 GPCRs adopt a two-step model for ligand binding and receptor activation^28^. However, this may not apply to small-molecule modulators that bind to a small part of the peptide-binding pocket or even to an allosteric site, thus bypassing the reorganization of ECD, such as PCO371, a small-molecule agonist bound within the intracellular pocket of 7 class B1 GPCRs to directly interact with G_s_ protein^6,7^. These atypical activation processes indicate that intracellular signal transducers may have opportunity to engage the receptor without extracellular stimuli, inducing basal activity. A previous study has demonstrated that constitutive cAMP signaling from GLP-1R, in the absence of GLP-1 or GCG, contributed to glucose-induced insulin secretion, indicating the physiological relevance of basal signaling activity^29^.

The three cryo-EM structures of ligand-free G_s_-coupled GLP-1R, GCGR and GIPR presented here expanded our knowledge of G protein coupling and receptor activation from three aspects. First, the ligand-free receptors successfully promote a large outward movement of the cytoplasmic half of TM6 and reorganize the HETY and cytoplasmic polar networks to open an intracellular crevice for G protein coupling. Even though the overall G protein coupling interface is similar between the ligand-free and peptide-bound receptors, the former has weaker contacts with G_s_. Second, the extracellular halves of TMs 1, 6, and 7 of the ligand-free receptors are all structurally different from those of peptide-bound receptors in a receptor-specific manner. For instance, the ligand-free GIPR adopts a moderately kinked TM6 different from the fully active GIPR and the ligand-free GLP-1R or GCGR. Third, the TMD orthosteric pocket is partially occupied by ECLs in the ligand-free G_s_-coupled GCGR (via the inward stretched ECL2) and GIPR (via the inward folded TM6–ECL3) but not GLP-1R. Such special ECL2 and ECL3 conformations are not seen in other class B1 GPCRs. These results provide valuable structural evidence for the existence of a ligand-free and G_s_-coupled transitional state distinct from those reported previously, i.e., inactive (antagonist-bound and apo without G protein) and active (agonist-bound and agonist-bound plus G protein-coupled).

Structural superimposition of the ligand-free and G_s_-coupled receptor with that bound by peptide agonist reveals that agonist binding or G protein coupling individually can cause differential packing in a receptor-dependent manner but not to the extent shown in the fully active state^20^. Specifically, peptidic agonist (NNC0712 for GCGR and peptide 5 for GLP-1R) binding reshapes the orthosteric pocket but cannot appropriately reorganize the central polar network to propagate extracellular signals^30,31^. As a result, the sharp kink in TM6 could not be formed and the intracellular crevice is still closed. In comparison, the structures of ligand-free and G_s_-coupled GLP-1R, GCGR and GIPR suggest that G_s_ protein successfully not only opens the intracellular binding cavity without agonist action, but also rewires the extracellular orthosteric pocket to stabilize the receptor in a transitional state, ready for agonist binding. We noted that ECLs are constitutively flexible without agonist binding, thereby posing a conformation different from those observed in both active and inactive states. Although ECL3 of the ligand-free GLP-1R swings out of the peptide-binding pocket, the extracellular portion of TMD is maintained in a conformation close to the active state, because several residues located at the orthosteric site form an interaction network to stabilize the TMD core. However, GIPR has a uniquely folded TM6–ECL3 juncture covering the orthosteric site, and GCGR orthosteric site is partially occupied by ECL2, locking the receptors in a transitional state. Peptide binding tightens the orthosteric pocket and triggers conformational changes of L^6.48b^–Y^7.57b^–N^7.61b^–E^8.49b^–E392^GαH5^ motif, thereby transmitting signal from the extracellular to intracellular sides. Thus, despite different molecular features of the transitional receptors, agonist binding can reshape them to a similar conformational state that is required for receptor activation.

It is also possible that in the absence of ligand, the intracellular half of TMD can be independently mobilized by G_s_ protein, which in turn rearranges the extracellular half to form a transitional conformation. The receptor in this state, without an extracellular shutting conformation due to flexible ECD, may simultaneously mobilize the orthosteric pocket and ECLs (e.g., outward-splayed ECL3 of GLP-1R, inward-stretched ECL2 of GCGR and inward-folded ECL3 of GIPR) to facilitate the entry of the peptide N-terminus, leading to downstream signaling events. Previous studies indicate that agonist-bound intermediate and fully active states possess a higher activation energy barrier for GCGR than the class A GPCR adrenergic receptor^20^. It is therefore possible that the “transitional state” presented in our study could be a G_s_ pre-coupled status to cope with the energy barrier required for the opening of the intracellular end of TM6. This “G_s_-first” feature may allow more rapid activation than “agonist-first” model. Our findings would certainly broaden the overall understanding of activation mechanisms of class B1 GPCRs and provide insights into the development of better therapeutics targeting GLP-1R, GCGR and GIPR.

## Materials and methods

### Constructs

The human GLP-1R, GCGR and GIPR were cloned into pFastBac vector and modified with their native signal sequences replaced by the HA signal peptide to facilitate receptor expression. The NanoBiT tethering strategy was used for the complexes as described previously^32^, in which the receptor C-terminus was directly attached to LgBiT subunit followed by a TEV protease cleavage site and a double maltose binding protein (MBP) tag. To improve the thermostability of GCGR–G_s_ complex, 45 residues (H433–F477) were truncated and the affinity tag HPC4 was added to the receptor C-terminus. For GIPR, the residue T345 was mutated to phenylalanine, a BRIL fusion protein was added to the N-terminus with 2GSA linker, and 45 amino acids (Q422–C466) at C-terminus were truncated. These modifications did not alter receptor pharmacology^15,18^. An engineered G_s_ construct (G112) was used for expression and purification of the GLP-1R–G_s_ or GCGR–G_s_ complexes^33^. A dominant-negative Gα_s_ (DNGα_s_) with 9 mutations (S54N, G226A, E268A, N271K, K274D, R280K, T284D, I285T and A366S) were generated by site-directed mutagenesis to stabilize interactions with the βγ subunits in the GIPR–G_s_ complex^34^. Rat Gβ1 was cloned with a C-terminal SmBiT (peptide 86 or HiBiT, Promega) connected to a 15-amino acid polypeptide linker^35^. The modified rat Gβ1 and bovine Gγ2 were both cloned into pFastBac vector. Nanobody 35 (Nb35) with a C-terminal 6× His-tag was cloned into the expression vector (pET28a) and used to limit G protein dissociation by binding at Gα_s_–Gβ interface^36^.

### Complex expression and purification

*Spodoptera frugiperda* (Sf9) or High Five insect cells (Expression Systems) were grown in ESF 921 serum-free medium (Expression Systems) at 27 °C and 120 rpm. The Bac-to-Bac Baculovirus Expression System (Invitrogen) was used to generate high-titer recombinant baculovirus for GLP-1R, GCGR, GIPR, and three G_s_ subunits. P0 viral stock was produced by transfecting 5 μg recombinant bacmids into Sf9 cells (2.5 mL, density of 1 × 10^6^ cells/mL) followed by 96 h incubation and then used to produce P1 and P2 baculoviruses. Cell culture was grown to a density of 3 × 10^6^ cells/mL and then infected with four separate baculoviruses, including the receptor and three G_s_ subunits. Complex formation and purification were performed as described previously^14^. For GLP-1R, the GLP-1R-LgBiT-2MBP, G112, Gβ1-HiBiT and Gγ2 were co-expressed by infecting Sf9 cells at a ratio of 1:1:1:1. For GCGR, the GCGR-LgBiT-2MBP, G112, His6-tagged Gβ1-HiBiT and Gγ2 were co-expressed by infecting Sf9 cells at a ratio of 1:3:3:3. For GIPR–G_s_ complex, BRIL-GIPR(T345F)-LgBiT-2MBP, DNGα_s_ (with 9 mutations), Gβ1-HiBiT and Gγ2 were co-expressed at a ratio of 1:3:3:3 in High Five cells. After 48 h incubation at 27 °C, the cells were collected by centrifugation and stored at –80 °C until use.

Cell pellets were lysed in a buffer containing 20 mM HEPES, pH 7.4, 50 mM NaCl and 2 mM MgCl_2_ supplemented with EDTA-free protease inhibitor cocktail (Bimake). Cell membranes were then collected by ultracentrifugation at 4 °C, 65,000× *g* for 35 min. A buffer consisting of 20 mM HEPES, 100 mM NaCl, pH 7.4, 10 mM MgCl_2_, 1 mM MnCl_2_, 100 μM TCEP, 25 mU/mL apyrase (Sigma-Aldrich), 15 μg/mL Nb35, protease inhibitor cocktail and 10% glycerol was used to resuspend the collected membranes. After incubating for 1 h at room temperature (RT), the complexes were solubilized from membrane using 0.5% (w/v) lauryl maltose neopentyl glycol (LMNG, Anatrace) and 0.03% (w/v) cholesteryl hemisuccinate (CHS, Anatrace) for 2 h at 4 °C. The supernatant was collected by centrifugation at 65,000× *g* for 30 min at 4 °C and then reacted with amylose resin for 2 h at 4 °C. After packing, the resin was washed with 20 column volumes of buffer containing 20 mM HEPES, pH 7.4, 100 mM NaCl, 10% (v/v) glycerol, 25 μM TCEP, 5 mM MgCl_2_, 1 mM MnCl_2_, 0.03% (w/v) LMNG, 0.01% (w/v) GDN and 0.008% (w/v) CHS. TEV enzyme was added to the resin and kept at 4 °C overnight to remove the 2× MBP tag. The complex was eluted from the resin and concentrated to 500 μL using a 100 kDa MWCO Amicon Ultra Centrifugal Filter. The size exclusion chromatography was carried out by loading the protein sample to Superdex 200 Increase 10/300GL or Superose 6 Increase 10/300GL (Cytiva) to obtain the monomer complex.

### Nb35 expression and purification

Nb35 was expressed in the periplasm of *E. coli* BL21 (DE3) and purified by nickel affinity chromatography as described previously^37^. Briefly, the Nb35 target gene was transformed into BL21 and grown in a TB culture medium with 100 μg/mL ampicillin, 2 mM MgCl_2_ and 0.1% (w/v) glucose at 37 °C, 180 rpm. The expression was induced by adding 1 mM IPTG when OD_600_ reached 0.7–1.2. The cell pellet was collected by centrifugation after overnight incubation. The HiLoad 16/600 Superdex 75 column (Cytiva) was used to separate the monomeric fractions with running buffer containing 20 mM HEPES, pH 7.5 and 100 mM NaCl. The purified Nb35 was flash-frozen in 30% (v/v) glycerol by liquid nitrogen and stored at –80 °C until use.

### Cryo-EM data acquisition

Cryo-EM samples were prepared by plunge vitrification in liquid ethane on a Vitrobot Mark IV (ThermoFisher Scientific) with blotting chamber set to 4 °C and 100% humidity. The purified samples at concentrations of 10–20 mg/mL were applied to glow-discharged holey carbon grids (Quantifoil, R1.2/1.3, Au 300 mesh) and blotted for 2.5 s, 3.5 s, and 3 s before plunging for the GLP-1R–G_s_–Nb35, GCGR–G_s_–Nb35 and GIPR–G_s_–Nb35 complexes, respectively. Data were collected on a Titan Krios (ThermoFisher Scientific) 300 kV electron microscope equipped with a Gatan K3 Summit direct electron detector and serial EM3.7 was used to acquire cryo-EM images. The microscope was operated at a nominal magnification of 46,685× in counting mode, corresponding to a pixel size of 1.071 Å. The total exposure time was set to 7.2 s with intermediate frames recorded every 0.2 s, resulting in an accumulated dose of 80 e/Å^2^ fractionated into a movie stack of 36 frames with defocus range of –1.2 μm to –2.2 μm. Totally, 4741 movies for GLP-1R−G_s_, 5553 movies for GCGR−G_s_ and 6517 movies for GIPR−G_s_ complexes were collected.

### Cryo-EM data processing

The collected data were processed by cryoSPARC (v3.2.0) as summarized in Supplementary Fig. S2. Motion correction and CTF estimation for micrographs were done by patch motion correction and patch CTF estimation, respectively. Micrographs under 5 Å CTF resolution were cut off by Curate Exposures, and particles were auto-picked by template picker referenced from a previously published map of GLP-1R−G_s_ complex (EMBD code: EMD-30867)^8^. Auto-picking yielded particle projections that were subjected to two rounds of reference-free 2D classification to discard false-positive particles or particles categorized in poorly defined classes. After 2D classifications, particles from better classes were selected and classified by hetero refinement into 6 classes using Ab-initio volume generated from 200,000 particles as reference model. Particles from the class with the clearest model were selected for further 3D classification where soft masks around G_s_ subunits and ECD were used in focused classifications to improve the density in these regions. After several rounds of hetero refinement, 3D reconstruction was performed by NU-refinement, and the final map was improved to 2.54 Å for GLP-1R–G_s_, 2.70 Å for GCGR–G_s_ and 2.86 Å for GIPR–G_s_ complexes. Reported resolution is based on the gold-standard Fourier shell correlation using the 0.143 criterion.

### Model building and refinement

The models of the GLP-1R–G_s_, GCGR−G_s_ and GIPR–G_s_ complexes were built based on the cryo-EM structures of GLP-1–GLP-1R–G_s_ (PDB: 6X18)^27^, glucagon–GCGR–G_s_ (PDB: 6LMK)^26^ and GIP–GIPR–G_s_ (PDB: 7DTY)^15^, respectively. The models were docked into relevant cryo-EM density maps using UCSF Chimera v1.15^38^, followed by iterative manual adjustment and rebuilding in COOT v0.9.2^39^. Real-space refinement was performed using Phenix v1.19.1^40^. The final refinement statistics were validated using the module comprehensive validation (cryo-EM) in Phenix v1.19.1^40^. The final refinement statistics are provided in Supplementary Table S1. Structural figures were prepared using UCSF Chimera X and PyMOL v2.1 (https://pymol.org/2/).

### Supplementary information

Supplementary information

## Data Availability

The atomic coordinates and the electron microscopy maps have been deposited in the Protein Data Bank (PDB) under the accession codes: 8WG7 (GLP-1R–G_s_–Nb35 complex), 8WG8 (GCGR–G_s_–Nb35 complex) and 8WA3 (GIPR–G_s_–Nb35 complex), and Electron Microscopy Data Bank (EMDB) with the accession codes: EMD-37504 (GLP-1R–G_s_–Nb35 complex), EMD-37505 (GCGR–G_s_–Nb35 complex) and EMD-37390 (GIPR–G_s_–Nb35 complex). All relevant data are available from the authors and/or included in the manuscript or Supplementary information.
