# Supplementary material for: Molecular features of the ligand-free GLP-1R, GCGR and GIPR in complex with Gs proteins
Source: Cell Discov. 2024 Feb 13;10:18. doi: 10.1038/s41421-024-00649-0 (PMC10861504; doi:10.1038/s41421-024-00649-0)
Supplement: Supplementary file 1 — Supplementary information [file 41421_2024_649_MOESM1_ESM.pdf]

Supplementary Information

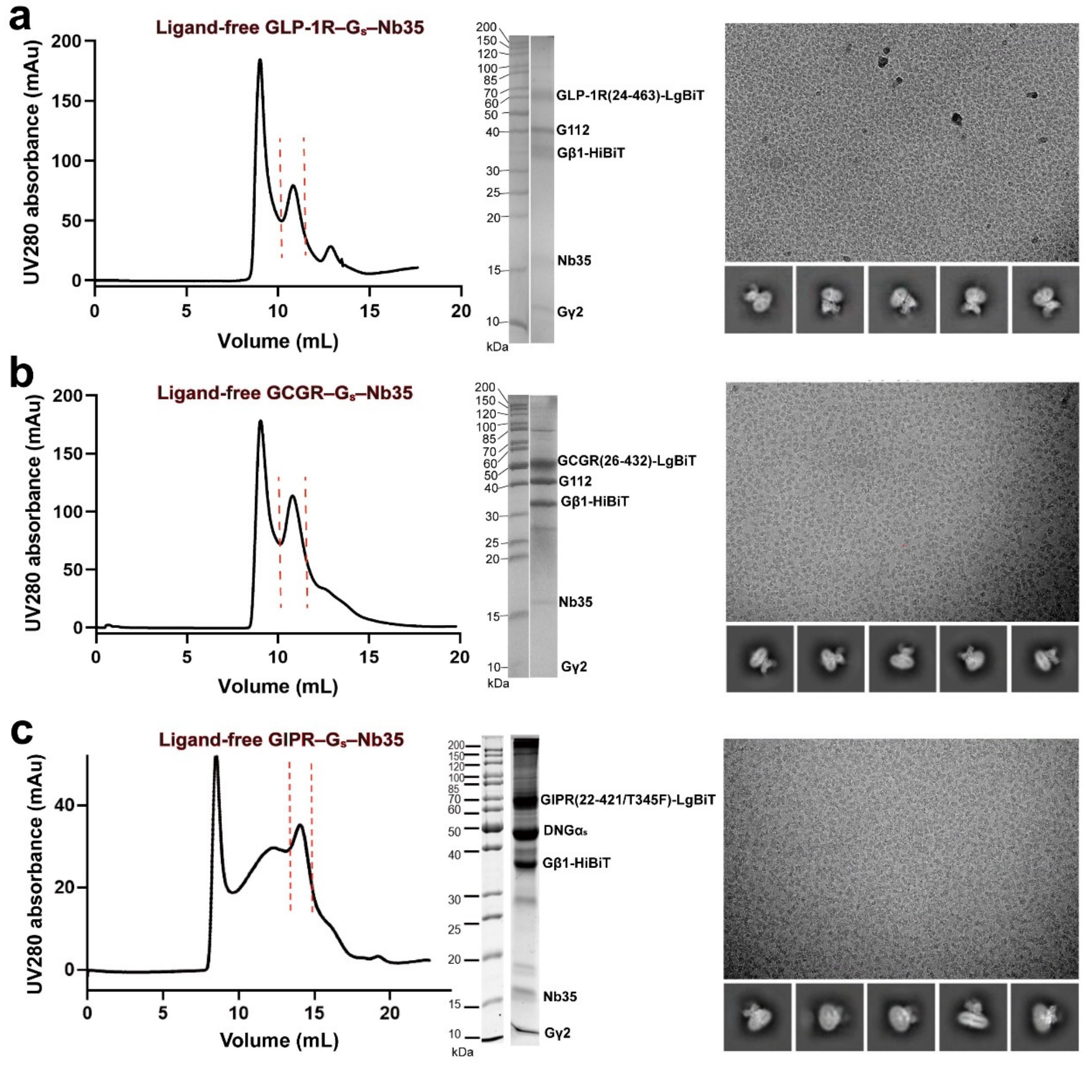

Supplementary Figure S1. Purification and cryo-EM imaging of the GLP-1R–Gs (a), GCGR–Gs (b) and GIPR–Gs (c) complexes. Left, analytical size-exclusion chromatography and corresponding SDS-PAGE/Coomassie blue. Fractions of the protein complexes are shown by red dashed lines; Right, representative cryo-EM micrograph and two-dimensional class averages of the selected particles.

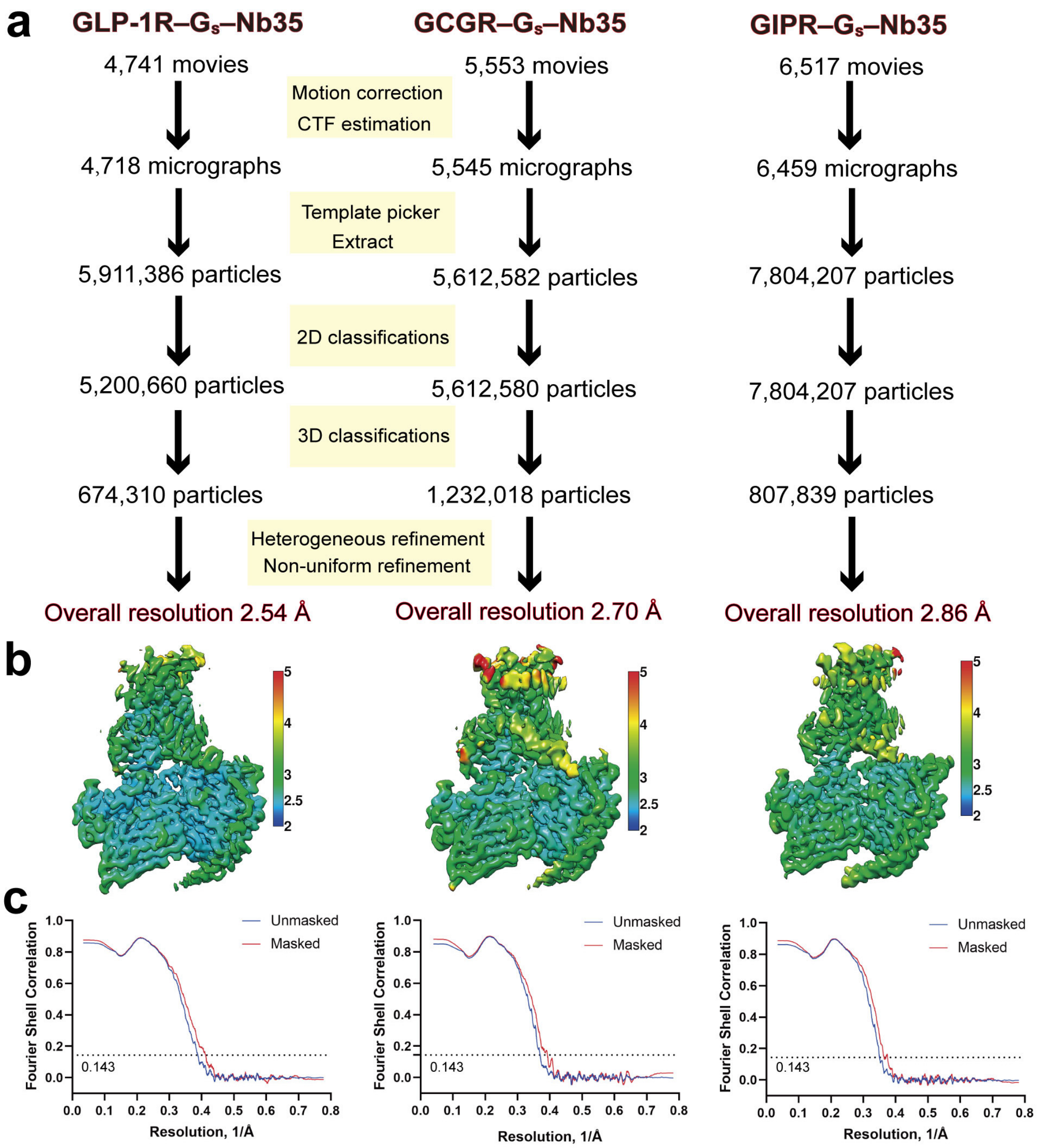

Supplementary Figure S2. Cryo-EM structure determination of the GLP-1R–G$_s$, GCGR–G$_s$ and GIPR–G$_s$ complexes. a Cryo-EM data processing flow chart. **b** Density map colored by local resolution. **c** Gold standard Fourier shell correlation (FSC) curves of overall refined structures, indicating the global resolution at 0.143 FSC threshold.

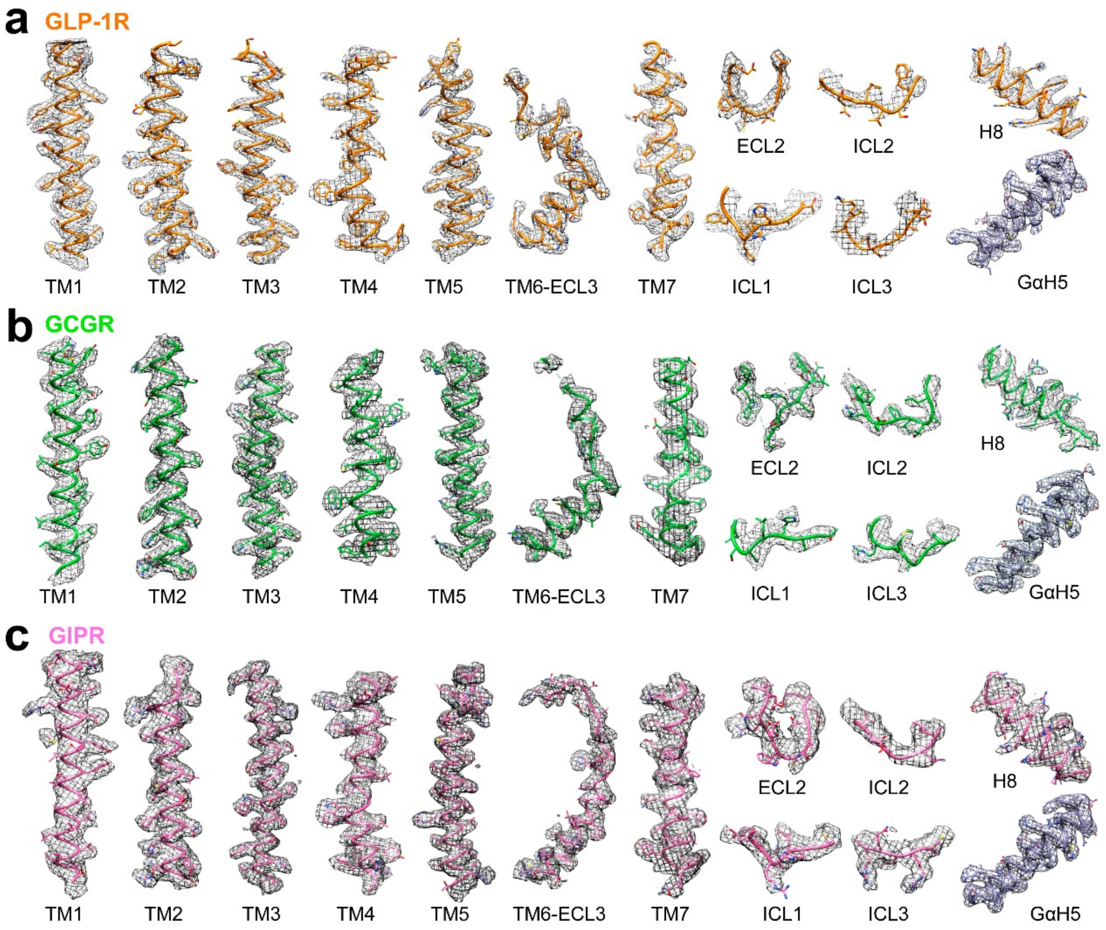

Supplementary Figure S3. Cryo-EM density maps and models for the GLP-1R–G_s (a), GCGR–G_s (b) and GIPR–G_s (c) structures. The cryo-EM density maps and models are shown for all seven transmembrane helices (TMs), extracellular loops 2 and 3 (ECL2 and ECL3), intracellular loops 1-3 (ICLs 1-3) and helix 8 (H8) of the receptor, and α5 helix of G_s protein (GαH5).

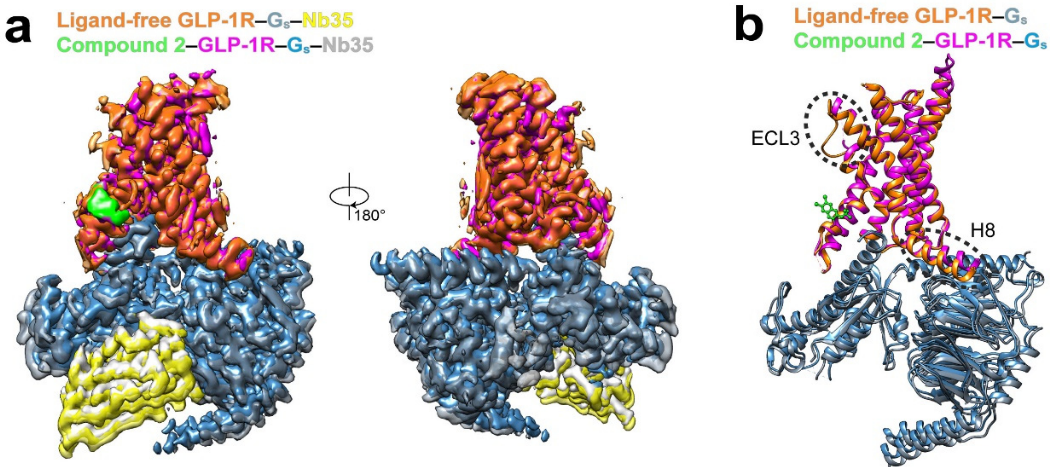

Supplementary Figure S4. Comparison of the ligand-free and compound 2-bound GLP-1R structures. a Superimposition of maps of the ligand-free and compound 2-bound GLP-1R (EMDB: EMD-31329) in complex with G_s protein shows an overall similarity. **b** Superimposition of the ligand-free and compound 2-bound GLP-1R (PDB ID: 7EVM) structures shows the conformational changes of ECL3 and H8 induced by compound 2.

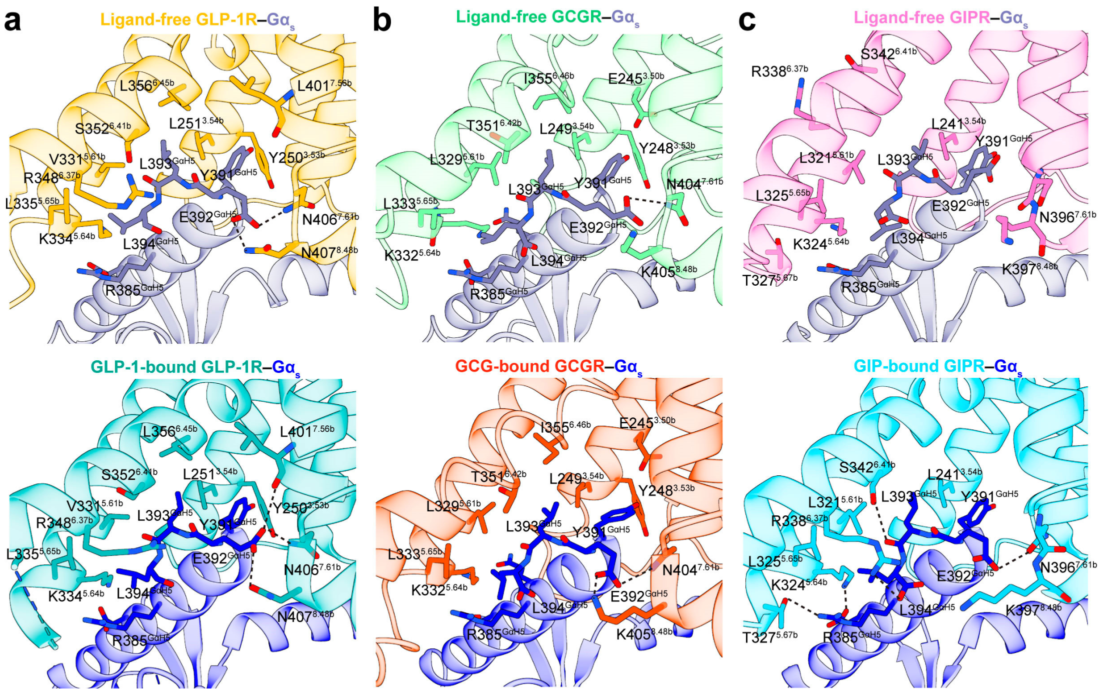

Supplementary Figure S5. Comparison of the binding mode of C-terminal GαH5. The C terminus of GHα5 forms different interactions with the receptor core of the ligand-free or endogenous ligand-bound GLP-1R **(a)**, GCGR **(b)** and GIPR **(c)**. The interacting residues are shown as sticks. H-bonds are shown as black dashed lines. PDB IDs: GLP-1-bound GLP-1R (6X18), GCG-bound GCGR (6LMK) and GIP-bound GIPR (7DTY). The position of $N^{7.61b}$ in the figure refers to $N^{8.47b}$ in GPCRdb numbering.

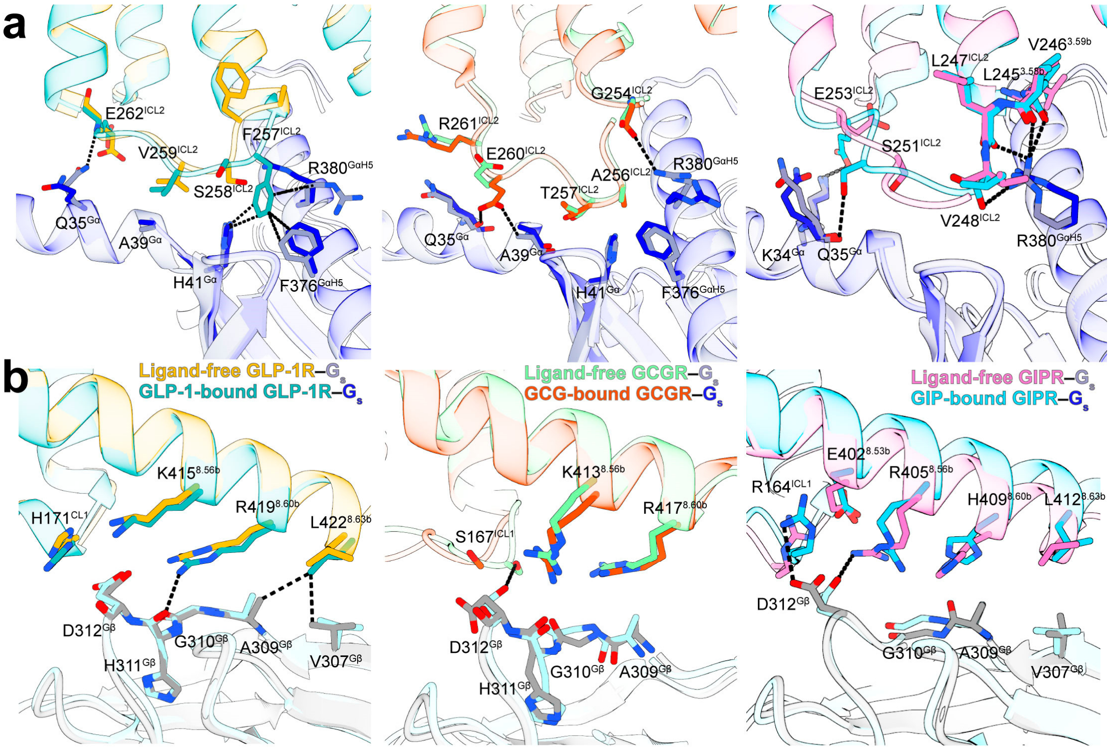

Supplementary Figure S6. Comparison of the interface between ICL2 (a) or helix 8 (b) and G_s in ligand-free and peptide-bound GLP-1R, GCGR and GIPR. a ICL2 forms less hydrophobic interactions with residues in α5 and αN helixes of $G\alpha_s$ in ligand-free complexes. **b** Helix 8 forms less interactions with Gβ. Key interacting residues are shown as sticks. Different interactions are shown as black dashed lines. PDB IDs: GLP-1-bound GLP-1R (6X18), GCG-bound GCGR (6LMK) and GIP-bound GIPR (7DTY).

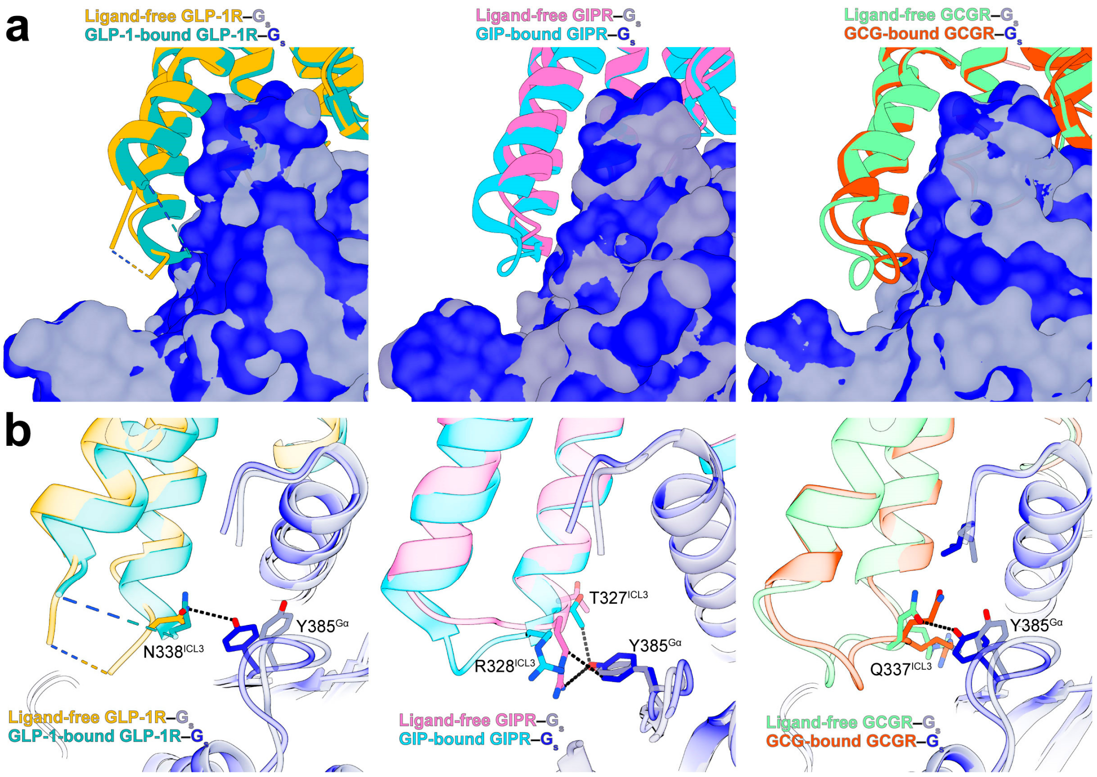

Supplementary Figure S7. Comparison of the interface between ICL3 and Gα_s in ligand-free and peptide-bound GLP-1R, GCGR and GIPR complexes. **a** ICL3 points to a cavity formed by αN-β1 hinge and α5 helix region of Gα_s in different poses. **b** Residue Y385$^{G\alpha}$ forms polar interactions with ICL3 in the ligand-bound GLP-1R, GCGR and GIPR structures. Key interacting residues are shown as sticks. Different interactions are shown as black dashed lines. PDB IDs: GLP-1-bound GLP-1R (6X18), GCG-bound GCGR (6LMK) and GIP-bound GIPR (7DTY).

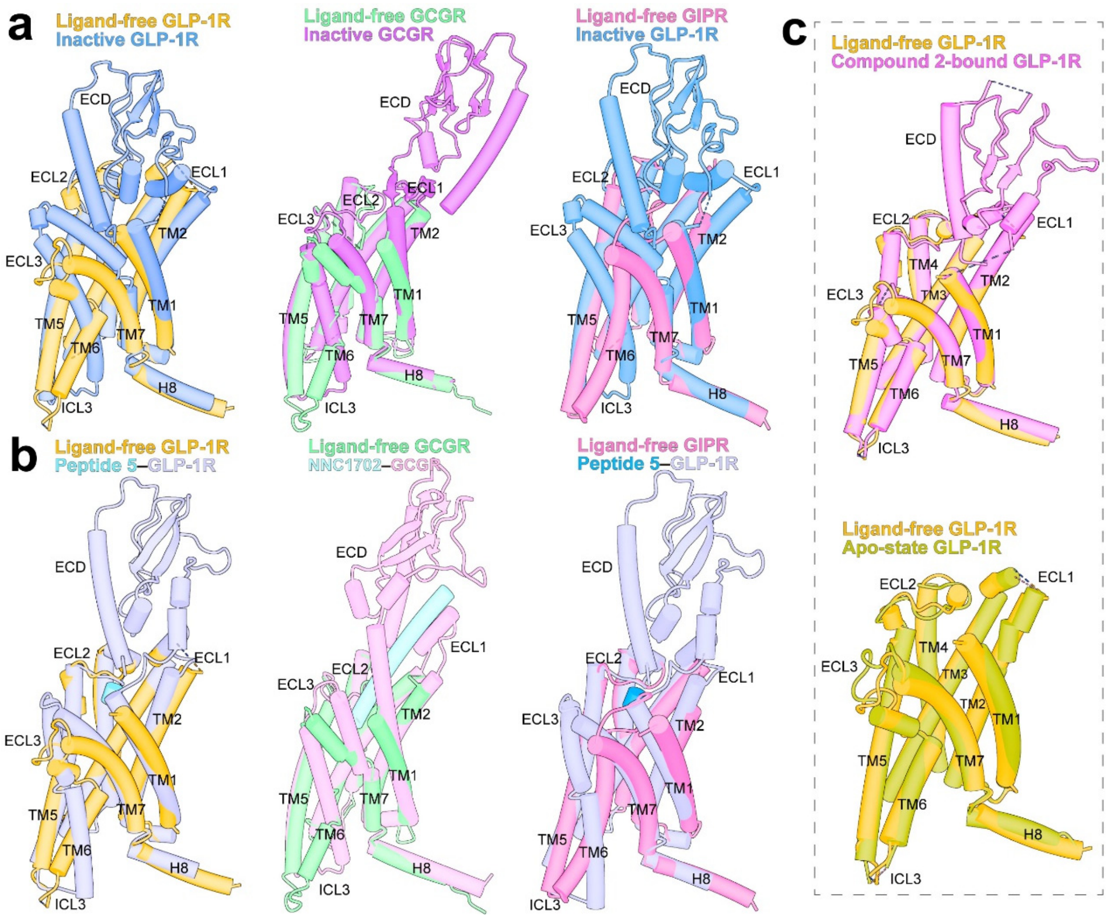

Supplementary Figure S8. Conformational comparison of ligand-free GLP-1R, GCGR and GIPR with their counterparts in inactive or intermediate state. a Superimposition of ligand-free GLP-1R, GCGR, GIPR structures and inactive state GLP-1R (PDB ID: 6LN2) or GCGR (PDB ID: 5XEZ) shows the conformational changes of TMD bundles induced by G_s coupling. **b** Superimposition of ligand-free GLP-1R, GCGR, GIPR structures and intermediate state GLP-1R (PDB ID: 5NX2) or GCGR (PDB ID: 5YQZ) shows the conformational changes of TMD bundles induced by agonist binding. **c** Ligand-free GLP-1R is superimposed onto that bound by compound 2 (PDB ID: 7DUR) and the apo GLP-1R (PDB ID: 7RG9), exhibiting a distinct ECL3 conformation.

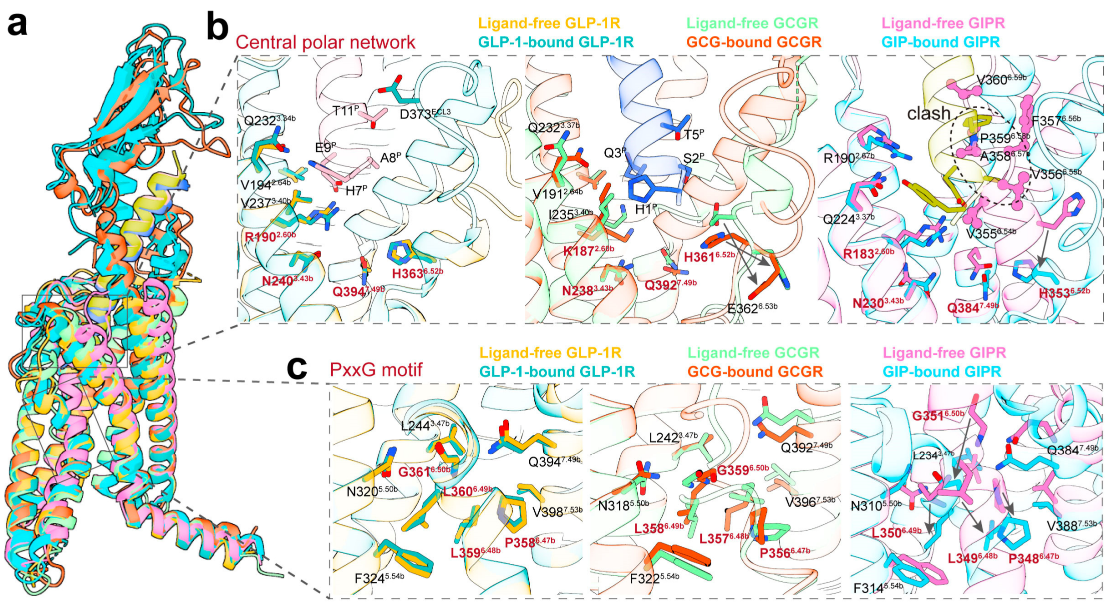

Supplementary Figure S9. Illustration of conformational changes triggered by peptide binding. a Superimposed structures of ligand-free and peptide-bound receptors. **b** Close-up views of conformational changes in the conserved central polar network (R$^{2.60b}$–N$^{3.43b}$–H$^{6.52b}$–Q$^{7.49b}$). N terminus of GIP clashes with TM6 (dashed circle). **c** Close-up views of conformational changes for the conserved PxxG active switch (P$^{6.47b}$–L$^{6.48b}$–L$^{6.49b}$–G$^{6.50b}$). Gray arrows represent major conformational changes. PDB IDs: GLP-1-bound GLP-1R (6X18), GCG-bound GCGR (6LMK) and GIP-bound GIPR (7DTY).

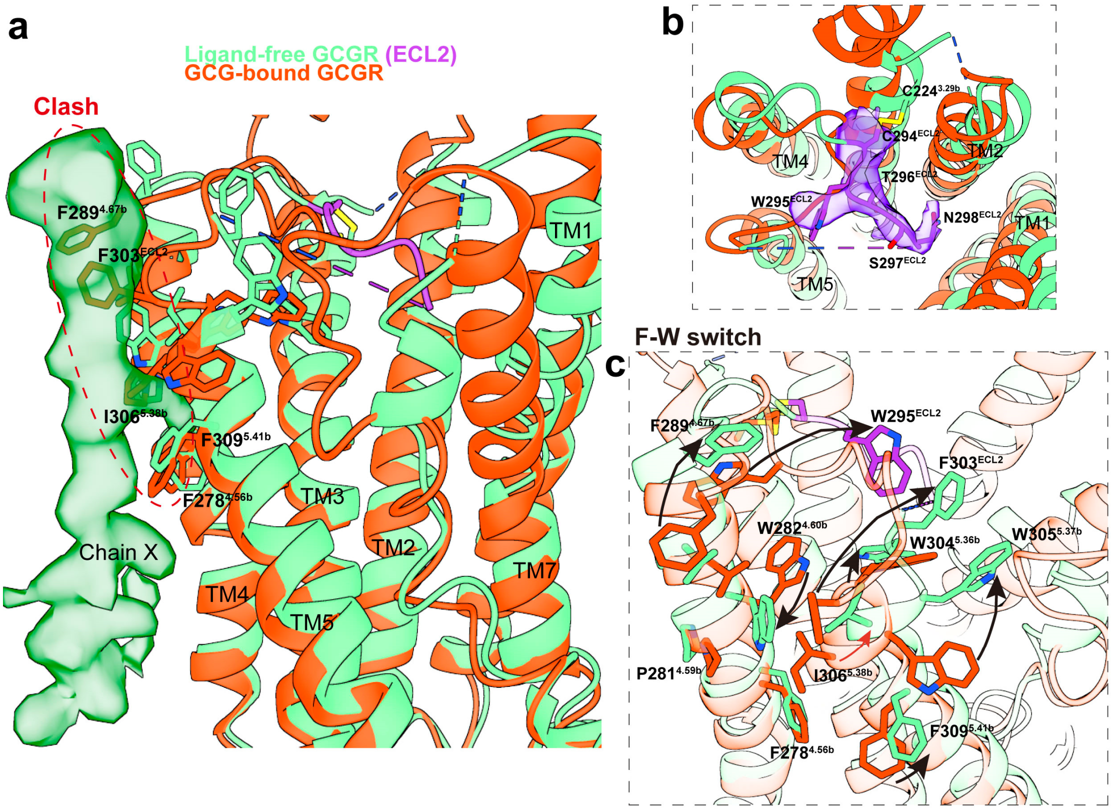

Supplementary Figure S10. Chain X-induced conformational changes in the ligand-free GCGR structure. a The binding position of chain X (cryo-EM density in green) clashes with the juxtamembrane region of TM4 and TM5. **b** A top view of the GCGR structure shows that the ECL2 (cryo-EM density in purple) inserted to the orthosteric binding pocket. **c** Superimposed structures of ligand-free and GCG-bound GCGR (PDB ID: 6LMK) show the conformational changes induced by chain X. Black arrows indicate representative conformational changes involving residues F281, W282, F289, W295, F303, W304, W304, and F309, named "F-W" switch here.

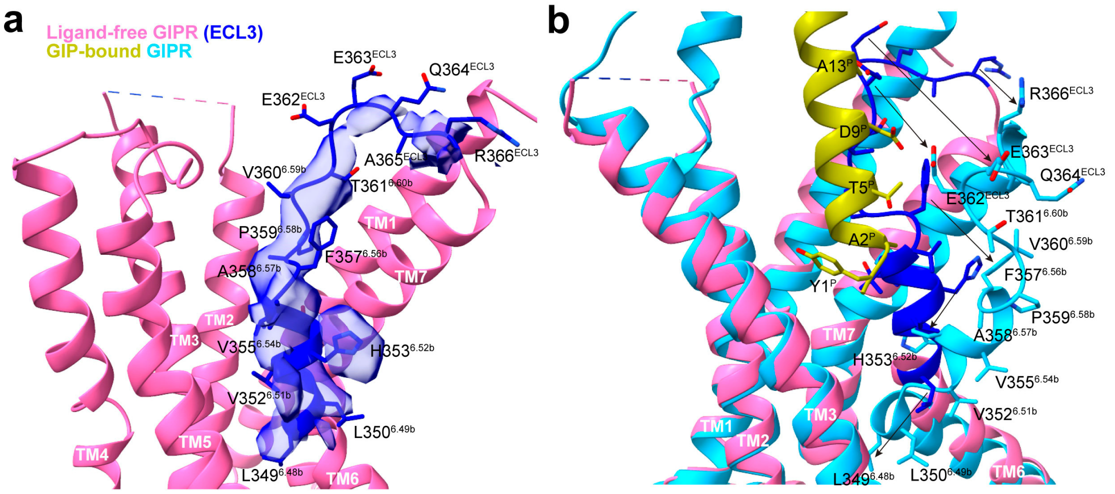

Supplementary Figure S11. Analysis of ECL3 in the ligand-free GIPR structure. a A side view of the GIPR structure shows that the TM6–ECL3 junction (cryo-EM density in blue) inserted to the orthosteric binding pocket. **b** Superimposition of ligand-free and GIP-bound GIPR (PDB ID: 7DTY) structures shows the conformational changes of ECL3 induced by GIP binding. Black arrows indicate representative conformational changes.

Supplementary Table S1. Cryo-EM data collection, refinement and validation statistics.

	**GLP-1R–G_s complex**	**GCGR–G_s complex**	**GIPR–G_s complex**
**Data collection and processing**			
Magnification	46,685	46,685	46,685
Voltage (kV)	300	300	300
Electron exposure (e^{-}/Å^2)	80	80	80
Defocus range (μm)	-1.2 to -2.2	-1.2 to -2.2	-1.2 to -2.2
Pixel size (Å)	1.071	1.071	1.071
Symmetry imposed	C1	C1	C1
Final particle images (no.)	674,310	1,232,018	807,839
Map resolution (Å)	2.54	2.70	2.86
FSC threshold	0.143	0.143	0.143
**Refinement**			
Initial model used (PDB ID)	6X18	6LMK	7DTY
Model resolution (Å)	2.5	2.7	2.9
Model composition			
Non-hydrogen atoms	8,123	8,044	8,163
Protein residues	1,030	1,037	1,031
Lipids	0	0	0
R.m.s. deviations			
Bond lengths (Å)	0.003	0.003	0.004
Bond angles (°)	0.618	0.655	0.755
**Validation**			
MolProbity score	1.74	1.77	1.97
Clash score	9.62	8.47	10.33
Poor rotamers (%)	1.16	1.30	1.37
Ramachandran plot			
Favored (%)	96.94	96.56	95.17
Allowed (%)	3.06	3.44	4.83
Disallowed (%)	0	0	0
